# Observation on the efficacy of combined electrical stimulation in the treatment of incomplete spinal cord injury: a randomized controlled trial

**DOI:** 10.3389/fneur.2026.1774055

**Published:** 2026-04-24

**Authors:** Xiaolei Chu, Rui Sun, Shuang Han, Zikang Yang, Ying Bai, Sining Zhao, Jingran Yuan, Zheng Xing, Wei Wang, Lei Zhang, Qi Li

**Affiliations:** 1 Department of Rehabilitation, Tianjin University Tianjin Hospital, Tianjin, China; 2 Haihe Laboratory of Brain–Computer Interaction and Human–Machine Integration, Tianjin, China; 3 School of Medical Technology, Tianjin University of Traditional Chinese Medicine, Tianjin, China; 4 Tianjin Key Laboratory of Exercise Physiology and Sports Medicine, Institute of Sport Exercise and Health, Tianjin University of Sport, Tianjin, China; 5 Department of Medical, Tianjin University, Tianjin, China

**Keywords:** bioinformatics analysis, cluster analysis, electrical stimulation, Electro Acupuncture, incomplete spinal cord injury

## Abstract

**Objective:**

This research evaluates the effectiveness of combined electrical stimulation therapy in patients with incomplete spinal cord injury.

**Methods:**

Twenty-five eligible patients from Tianjin Hospital’s Rehabilitation Department were randomly divided into an experimental group (*n* = 13) and a control group (*n* = 12). The experimental group received combined electrical stimulation, while the control group had conventional needle electrode therapy. Both groups were treated 5 times weekly for 30 min each session over 8 weeks. Sensory and motor functions were evaluated using the ASIA scales, and the BioNeuro Infiniti system measured RMS values and integrated electromyography.

**Results:**

Post-treatment, the experimental group demonstrated improved sensory and motor functions, functional independence, and biochemical blood markers compared to the control group (*p* < 0.05).

**Conclusion:**

Both combined electrical stimulation and needle electrode therapy effectively treat incomplete spinal cord injuries over 8 weeks, with combined electrical stimulation showing greater efficacy.

**Clinical trial registration:**

https://www.chictr.org.cn/showproj.html?proj=129549, identifier ChiCTR2100052017.

## 1 Background

Spinal cord injury (SCI) is a severe central nervous system trauma that can disrupt sensorimotor circuits, leading to impaired motor, sensory, and autonomic functions, often causing permanent paralysis below the injury. Research indicates SCI occurs more frequently in developed countries (1, 2). Traffic accidents and falls are leading causes of spinal cord injuries (SCI), particularly in males under 50, creating major economic and social challenges for families and society (3). SCI not only harms the spinal cord but also severely impacts the brain and peripheral nervous system. Treatment typically involves medications, hyperbaric oxygen therapy, and invasive epidural stimulation (4). Current treatments for SCI have limitations: drugs are non-specific with side effects and unsuitable for long-term use; hyperbaric oxygen therapy is mainly effective only in the acute phase with an unclear mechanism; and epidural stimulation is costly and risky.

Thus, there is a need for more effective therapies and a comprehensive approach to SCI rehabilitation. A key challenge in SCI recovery is stimulating neural network activation, encouraging axonal regeneration, and enhancing the nerve regeneration environment. Electrical stimulation, a widely used and cost-effective therapy in neural rehabilitation, is known for its simplicity and minimal side effects (5). Effective electrical stimulation therapies for SCI recovery are categorized by stimulation sites: head, spinal cord, and peripheral nerves.

Multiple studies have demonstrated that Electro Acupuncture and similar treatments have achieved significant efficacy in improving bladder function, motor function, and cognitive impairment after SCI (6–10). A combined electrical stimulation system was developed to reconstruct sensorimotor pathways after SCI (11); High-frequency electrical epidural stimulation has been shown to reduce spasticity and help restore lower limb motor function in patients with SCI (12). A study of incomplete SCI long-terms followed up showed that patients who received electrical stimulation recovered better in sensory function, muscle spasticity, muscle strength, and bowel function than control group (13). The combination of multiple electrical stimulation for SCI also has good efficacy. A prospective interventional study conducted combined with electrical stimulation in patients with incomplete SCI who chose a combination of central and peripheral electrical stimulation, which was proven to be effective in improving upper limb motor function in patients with incomplete SCI (14). Although the combination of electrical stimulation is effective, further research is needed to optimize the treatment regimen—to conduct it in a safer and more effective way for patients.

While each method is somewhat effective, combining them yields better outcomes, though limitations and controversies remain. We suggest using acupuncture needles for electrical stimulation instead of implanted electrodes to lower costs, eliminate surgical risks, and accurately target stimulation. Combining electrical stimulation of the head, spinal cord, and peripheral nerves activates the motor cortex, aids nerve regeneration at the spinal injury site, and stimulates ascending nerve pathways. We use portable ultrasound to accurately locate the spinal epidural and sciatic nerve before electrical stimulation, ensuring precise acupuncture needle insertion and minimizing harm. This method activates the entire nervous system, aiding neural recovery.

## 2 Methods

### 2.1 Participants and grouping

This randomized controlled trial involved 25 patients from Tianjin Hospital’s Rehabilitation Department who met the study’s inclusion criteria. Patient recruitment for this research was conducted from Dec 1, 2021 to Mar 1, 2024. They were randomly divided into an experimental group (*n* = 13) and a control group (*n* = 12).

#### 2.1.1 Inclusion criteria

(1) Patients must have a clinical diagnosis of incomplete SCI, as determined by clinical signs and imaging modalities such as X-ray, CT, and MRI, and be classified as grade B to D on the American Spinal Injury Association (ASIA) Impairment Scale, with accompanying lower limb dysfunction;
(2) The duration of the condition must range from 1 to 6 months;
(3) Patients must have undergone laminectomy and decompression surgery subsequent to the SCI, with stable vital signs postoperatively;
(4) Patients or their legal representatives must have a comprehensive understanding of the study procedures and must have voluntarily provided informed consent by signing the appropriate documentation.

#### 2.1.2 Exclusion criteria

(1) Serious conditions affecting neurological assessment, like fractures or severe brain injuries;
(2) Unstable vital signs or communication-impairing cognitive/psychiatric disorders;
(3) Persistent spasticity with an Ashworth Scale score of 2–4;
(4) Severe systemic conditions affecting heart, liver, kidney, or blood.

### 2.2 Treatment protocol

#### 2.2.1 Combined electrical stimulation

(1) Stimulation Sites: In a prone position, the patient received simultaneous electrical stimulation to the head, spinal cord, and peripheral nerves.

 (a) Electrical Stimulation of the Head: Stimulation targeted the motor cortex, 1.5 cm in front of the central sulcus. Acupuncture needles were placed at 1/5 of the distance along the bilateral precentral gyrus, connected to an electrical stimulator, and then activated. The equipment of electrical stimulation has been approved by National Medical Product Administration for the treatment of SCI.
 (b) Ultrasound-Guided Spinal Cord Stimulation:

Literature on acupuncture—moxibustion treatment of SCI from database inception to Oct 1, 2024 was retrieved from Wanfang, CNKI, SinoMed, VIP, and PubMed. In Chinese databases, subject—term search with terms like “acupuncture,” “spinal cord injury” was used; in English ones, a combination of MeSH and free terms such as “acupuncture,” “spinal cord injury” was adopted.

Inclusion criteria:

(1) Randomized controlled clinical trials.
(2) Subjects with diagnosed SCI.
(3) Research centered on acupuncture—moxibustion, used alone or with other therapies.
(4) Standard acupuncture techniques, recognized diagnostic and efficacy criteria, and demonstrated effectiveness.

Exclusion criteria:

(1) Reviews, animal experiments, systematic reviews, and meta-analyses.
(2) Unclear acupuncture prescriptions.
(3) No control group or non-compliant control groups.

After screening, a database was built in Excel 2021. Acupoint names and meridians in prescriptions were standardized per relevant standards. Excel 2021 was used for descriptive analysis, SPSS Modeler 18.0 Apriori for association rule analysis, and SPSS Statistics 27.0 for hierarchical cluster analysis with dendrogram drawing.

**Figure 1:**
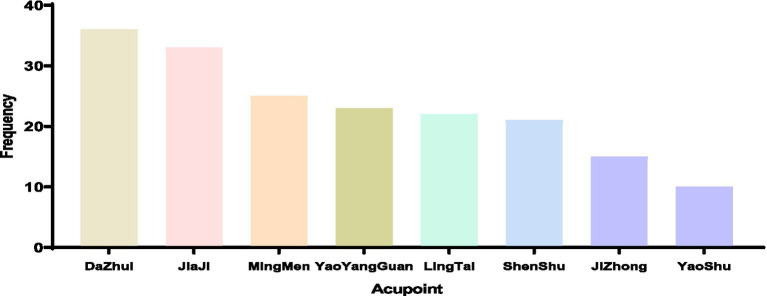
Frequency distribution of high-frequency acupoints in acupuncture treatment for SCI.

We have reviewed previous studies and identified the research methods to be used in this paper, which is part of the methodology. By reviewing previous articles, we identified methodological issues about acupuncture point selection. After screening, 52 articles were included, covering 70 acupoints used 467 times in total. Of these, 22 acupoints on the neck and back were used 214 times. For SCI treatment, the top three acupoints on the neck and back were Dazhui (36 times), Jiaji (33 times), and Mingmen (25 times). Eight acupoints were used at least 10 times, as shown in Figure 1.

Using the Apriori algorithm in SPSS Modeler 18.0, I analyzed association rules for 22 acupoints in an acupuncture prescription database. The support threshold was set at ≥25%, confidence at ≥85%, and a maximum of 2 antecedents. This resulted in 20 strong rules: 7 with 2 acupoints and 13 with 3. Notably, rules with ≥90% confidence focused on Dazhui, Jiaji, Lingtai, and Yaoyangguan acupoints. See Table 1 for details.

**Table 1:** Analysis of association rules of acupoints in the treatment of incomplete spinal cord injury by acupuncture.

Serial number	Consequent	Antecedent	Support (%)	Confidence (%)
1	Yaoshu	Jizhong	29.412	100.0
2	Yaoshu	Jizhong–Jiaji	25.49	100.0
3	Jiaji	Lingtai–Yaoyangguan	33.333	94.118
4	Dazhui	Lingtai–Yaoyangguan	33.333	94.118
5	Jiaji	Mingmen–Dazhui	29.412	93.333
6	Dazhui	Jizhong–Jiaji	25.49	92.308
7	Dazhui	Yaoyangguan	45.098	91.304
8	Jiaji	Lingtai	43.137	90.909
9	Dazhui	Lingtai	43.137	90.909
10	Dazhui	Lingtai–Jiaji	39.216	90.0
11	Jiaji	Lingtai–Dazhui	39.216	90
12	Dazhui	Yaoyangguan–Jiaji	39.216	90
13	Dazhui	Jiaji	64.706	87.879
14	Dazhui	Mingmen–Jiaji	31.373	87.5
15	Jiaji	Yaoyangguan	45.098	86.957
16	Jiaji	Jizhong	29.412	86.667
17	Jiaji	Jizhong–Yaoshu	29.412	86.667
18	Jizhong	Yaoshu–Jiaji	29.412	86.667
19	Dazhui	Yaoshu–Jiaji	29.412	86.667
20	Jiaji	Yaoyangguan–Dazhui	41.176	85.714

**Figure 2:**
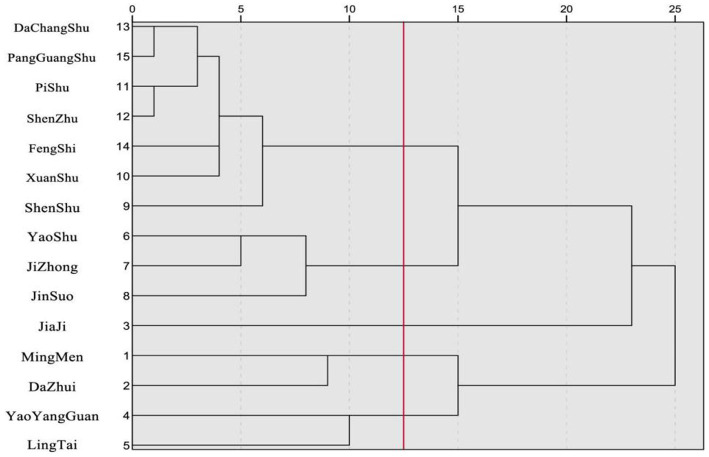
Dendrogram of cluster analysis for high-frequency acupoints in acupuncture treatment of incomplete SCI.

SPSS 27.0 was used for cluster analysis on the top 15 high-frequency acupoints using the between-groups linkage method and Pearson correlation coefficient. At a relative distance of 12.5, the acupoints showed good dispersion and close relationships. Excluding single-acupoint groupings, they were classified into four groups: (1) Shenshu (BL23), Dachangshu (BL25), Xuanshu (GV5), Fengshi (GB31); (2) Yaoshu (GV2), Jizhong (GV6), Jinsuo (GV8); (3) Jiaji acupoints, Dazhui (GV14); (4) Yaoyangguan (GV3), Lingtai (GV10). See Figure 2 for details.

Dazhui (GV14) and Jiaji acupoints were chosen for spinal cord electrical stimulation to treat incomplete SCI due to their frequent use, strong correlations in the analysis, and potential to effectively aid recovery.

The Dazhui acupoint, found between the C7 and T1 vertebrae, is effective for cervical spinal cord injuries at C7 and above but less so for thoracic or lumbar injuries. In contrast, Jiaji acupoints, located on either side of the spine, are more effective for spinal cord injuries. Electrical stimulation of Jiaji acupoints near the injury site can enhance neural activity and promote recovery by activating the surrounding neural network. Clinical practice and research suggest selecting Jiaji acupoints above and below the SCI for acupuncture.

**Figure 3:**
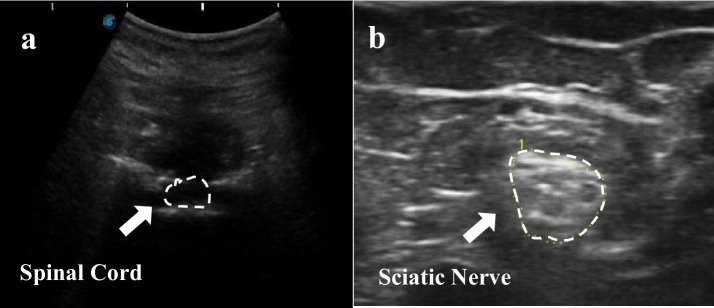
Ultrasonic localization of the spinal cord and sciatic nerve. (**a**. Spinal Cord; **b**. Sciatic Nerve).

Portable ultrasound was effectively used to accurately target the epidural space for spinal cord stimulation, ensuring precise needle placement and avoiding SCI. Post-laminectomy and decompression surgery, the epidural space was clearly visible on ultrasound. See Figure 3 for details. The procedure included:

Step1. Ultrasound identified the upper and lower planes of the injured spinal segment, marking the electrical stimulation points.

Step2. The distance from subcutaneous tissue to epidural dura mater was measured for each patient.

Step3. An acupuncture needle was inserted at the marked epidural site, following the measured depth, and connected to an electrical stimulator for stimulation.

**Figure 4:**
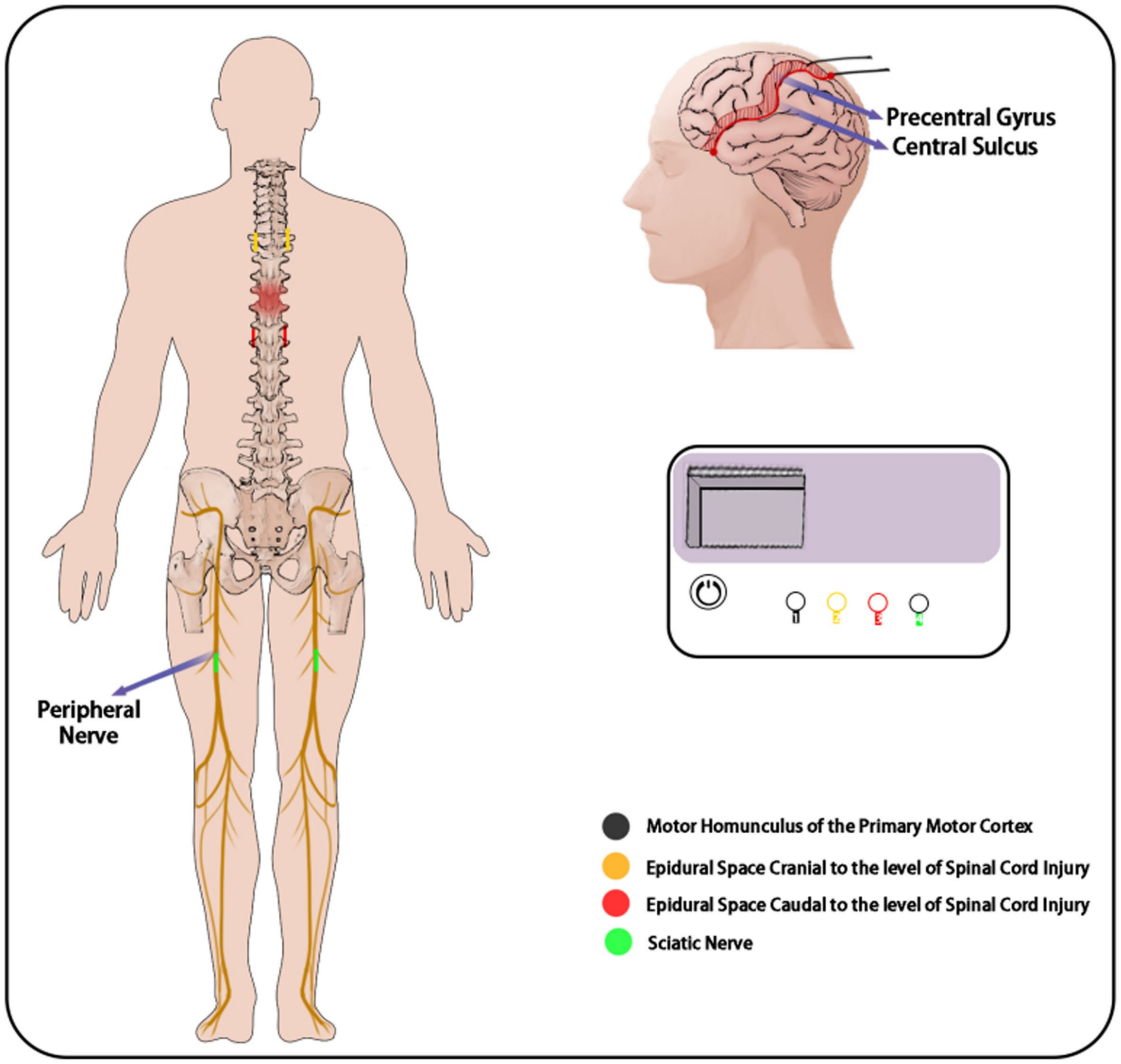
Schematic diagram of combined electrical stimulation.

 (c) Ultrasound-Guided Peripheral Nerve Stimulation: Peripheral nerve stimulation targeted the sciatic nerves on both sides. The sciatic nerve, the largest peripheral nerve, serves the lower limbs and extends from the lumbar-sacral spine through the gluteal area, posterior thigh, and calf to the heel. A portable ultrasound identified the sciatic nerve in the patient’s posterior thigh, and the distance from the skin to the nerve was measured (Figure 3). The needle was inserted at the marked site, connected to an electrical stimulator, and stimulated.

(2) Instruments

 (a) Hua Tuo SDZ-III Electronic Acupuncture Device;
 (b) Disposable acupuncture needles (0.3 mm x 40 mm, Beijing Keyuan Medical Instruments);
 (c) Kaili X5 Portable Ultrasound.

(3) Stimulation Parameters (15):

Intermittent wave, frequency 50 Hz, with gradually increasing current intensity to the tolerable level of the patient. See Figure 4 for details.

#### 2.2.2 Needle-type electrodes

The use of needle-type electrodes has been determined based on previous studies, and in fact, the use of electro-acupuncture has been shown to be effective in the treatment of incomplete SCI, with improvements in motor and sensory functions in patients (16–18).

(1) Location: The patient lay prone with the spinal injury exposed. The needle was inserted 0.5 cun (a unit in traditional Chinese medicine of acupuncture, which is approximately equal to 10–20 mm) lateral to the spinous process at the injury level, angled downward at 45 degrees for 0.5 cun, and connected to an electrical stimulator for stimulation.
(2) Instruments:
 (a) Hua Tuo SDZ-III Electronic Acupuncture Therapy Device;
 (b) Disposable acupuncture needles.
(3) Stimulation Parameters: Intermittent wave, frequency 100 Hz, with intensity adjusted to the patient’s tolerance or until rhythmic muscle contractions are observed.

#### 2.2.3 Conventional rehabilitation training included positioning, breathing exercises, muscle strength, balance, and standing/walking training

Both groups participated in this training. Additionally, the experimental group received combined electrical stimulation therapy, while the control group had needle-type electrode therapy. Each group underwent 30-min sessions of their respective therapies daily, 5 days a week, for 8 weeks.

### 2.3 Assessment metrics

#### 2.3.1 Functional independence scale

##### 2.3.1.1 Spinal Cord Independence Measure—Version 3 (SCIM-III)

The SCIM-III scale, comprising 17 items in self-care, respiration and sphincter management, and mobility, measures functional independence in SCI patients (19). Higher scores denote greater independence and improved quality of life. The total score is 100 points, with higher scores indicating better functional independence and lower scores reflecting greater effort or reliance on assistive devices.

##### 2.3.1.2 Functional Independence Measure (FIM)

The FIM scale, widely used in rehabilitation, assesses self-care and independence through motor (18 items) and cognitive (5 items) functions (20). Evaluators score patients based on independence, assistive device use, and assistance needed, with a total possible score of 126 and a minimum of 18. Assessments occur before and after 8 weeks of treatment, with higher scores reflecting greater independence and quality of life.

#### 2.3.2 Sensory function

Sensory function was measured using the ASIA sensory score scale before treatment and at 2, 4, 6, and 8 weeks after treatment (21). Evaluations covered 28 key sensory points on both sides of the trunk and limbs, testing light touch and pinprick sensation. Each side has a maximum score of 112, totaling 224 points bilaterally, with higher scores indicating better sensory function.

#### 2.3.3 Motor function

##### 2.3.3.1 ASIA motor function score

The ASIA motor function scale assessed motor function before treatment and at 2, 4, 6, and 8 weeks after treatment, focusing on the strength of 10 key muscle groups on each side. Muscles are rated from 0 to 5, with a maximum score of 100, where higher scores signify better motor function.

##### 2.3.3.2 Surface Electromyography (sEMG)

The study focused on sEMG testing of key lower limb muscles—rectus femoris, vastus medialis, vastus lateralis, tibialis anterior, and gastrocnemius—in patients with incomplete SCI and lower limb dysfunction, both before and after an 8-week treatment (22).

The SA7550 sEMG device from Thought Technology, Canada, used disposable Ag–AgCl electrodes. Three electrodes per muscle (two recording, one reference) were placed 2 cm apart. The patient lay supine as the evaluator explained the procedure for relaxation and familiarity. Key lower limb muscles were exposed for testing. To ensure accuracy, the evaluator cleaned electrode sites with alcohol swabs, removing hair and debris if necessary. Recording electrodes were placed 2 cm apart on the muscle belly, with a reference electrode on the tendon, ensuring proper skin contact to minimize errors.

Muscle and electrode placement:

(1) Rectus femoris: 2 cm above the midpoint of the iliac crest and the knee joint, aligned along the muscle fibers;
(2) Vastus medialis: Approximately 5 cm above the medial border of the patella, at the highest point of the muscle belly;
(3) Vastus lateralis: 15 cm above the lateral border of the patella, at the highest point of the muscle belly;
(4) Tibialis anterior: Located 1/4 to 1/3 of the distance between the knee and ankle joint, along the muscle fibers;
(5) Gastrocnemius: Distal to the knee joint, with electrodes placed at both the medial and lateral heads of the muscle belly.

Testing procedure: Initially, the patient relaxed for 20 s to record baseline signals. The therapist then passively stretched key lower limb muscles three times, holding each stretch for 20 s with a 10-s rest. The patient then performed maximal voluntary contractions for knee extension, ankle plantarflexion, and dorsiflexion, each for 20 s with a 10-s rest in between.

The system collected and analyzed EMG signals from electrodes, averaging three measurements. It recorded the Root Mean Square (RMS) and integrated electromyography (iEMG) values for each key muscle. RMS, a time-domain parameter, indicates the effective discharge value of the muscle, with higher values during voluntary contraction signifying greater muscle strength. iEMG represents the area under the processed EMG signal curve, showing active motor units and muscle fatigue resistance (23).

#### 2.3.4 Biochemical blood markers

Blood Sample Collection: Biochemical analyses of blood samples were performed by the Clinical Laboratory of Tianjin Hospital both prior to and following the 8-week treatment period (24).

**Figure 5:**
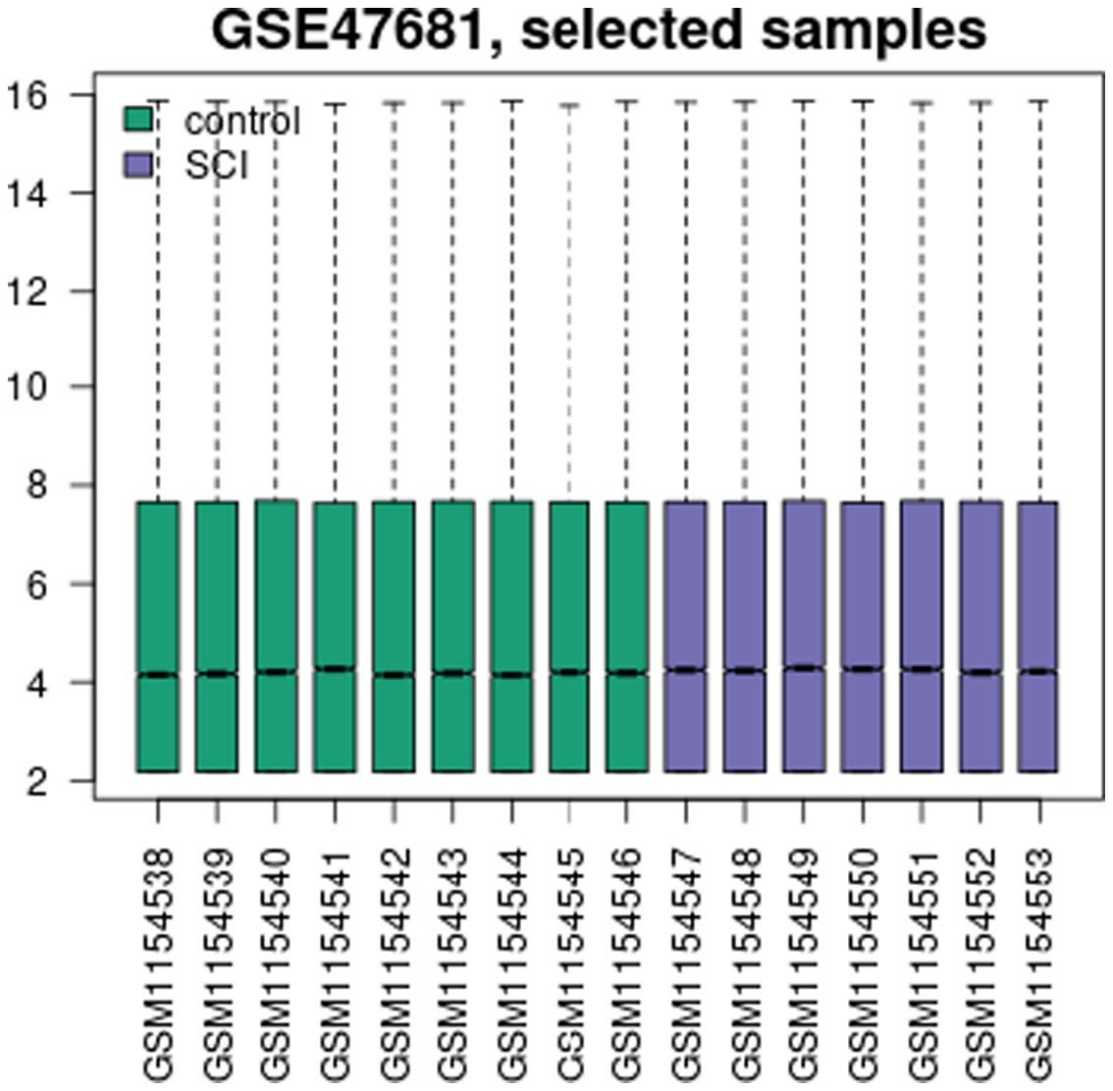
GSE47681 selected samples.

**Figure 6:**
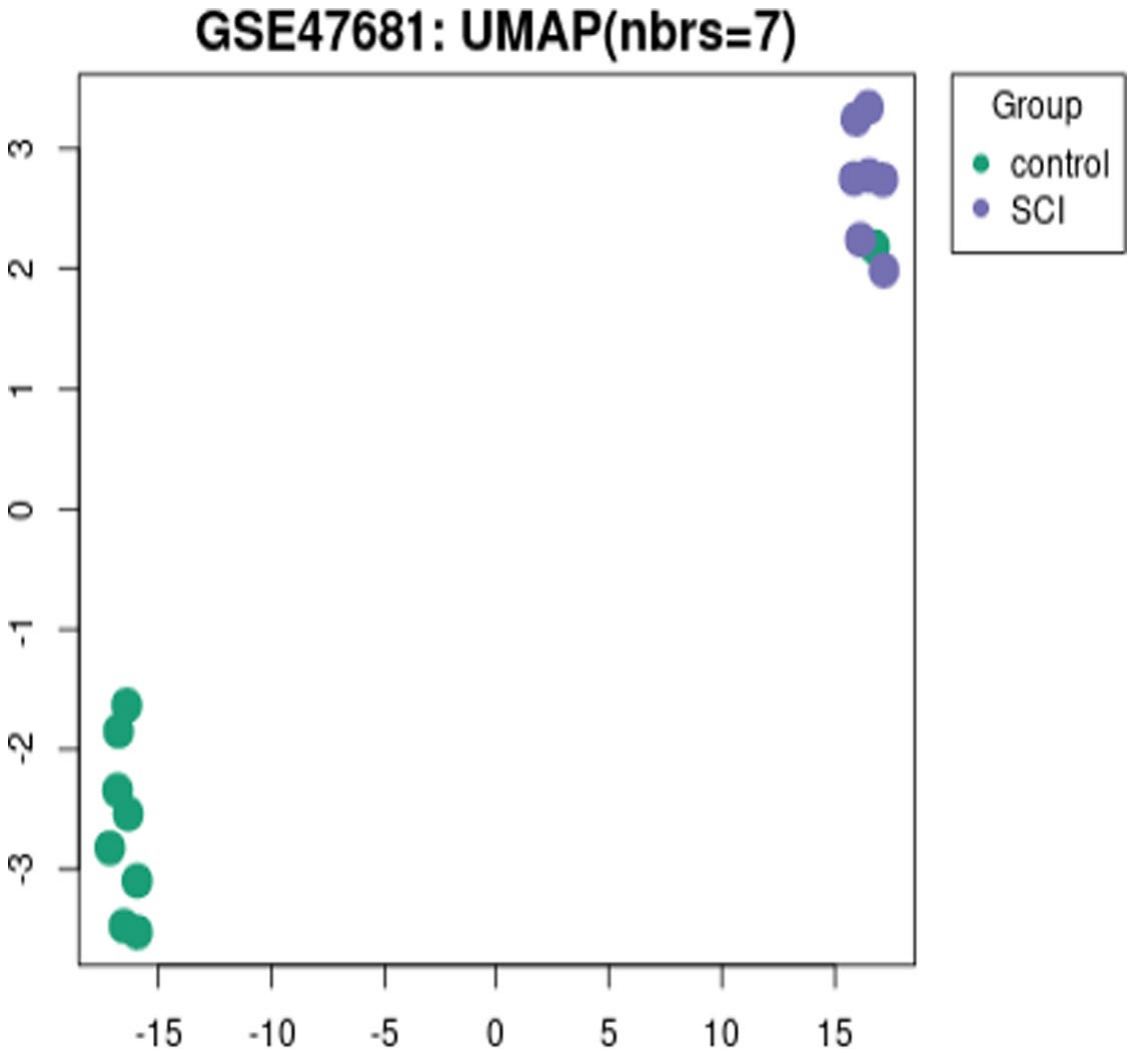
UMAP visualization of the control group and the SCI group.

The GSE47681 dataset was obtained from the GEO database. Figure 5 shows nearly level black lines in the box plot. UMAP analysis, depicted in Figure 6, reveals clear clustering and significant differences between the Control and SCI groups.

**Figure 7:**
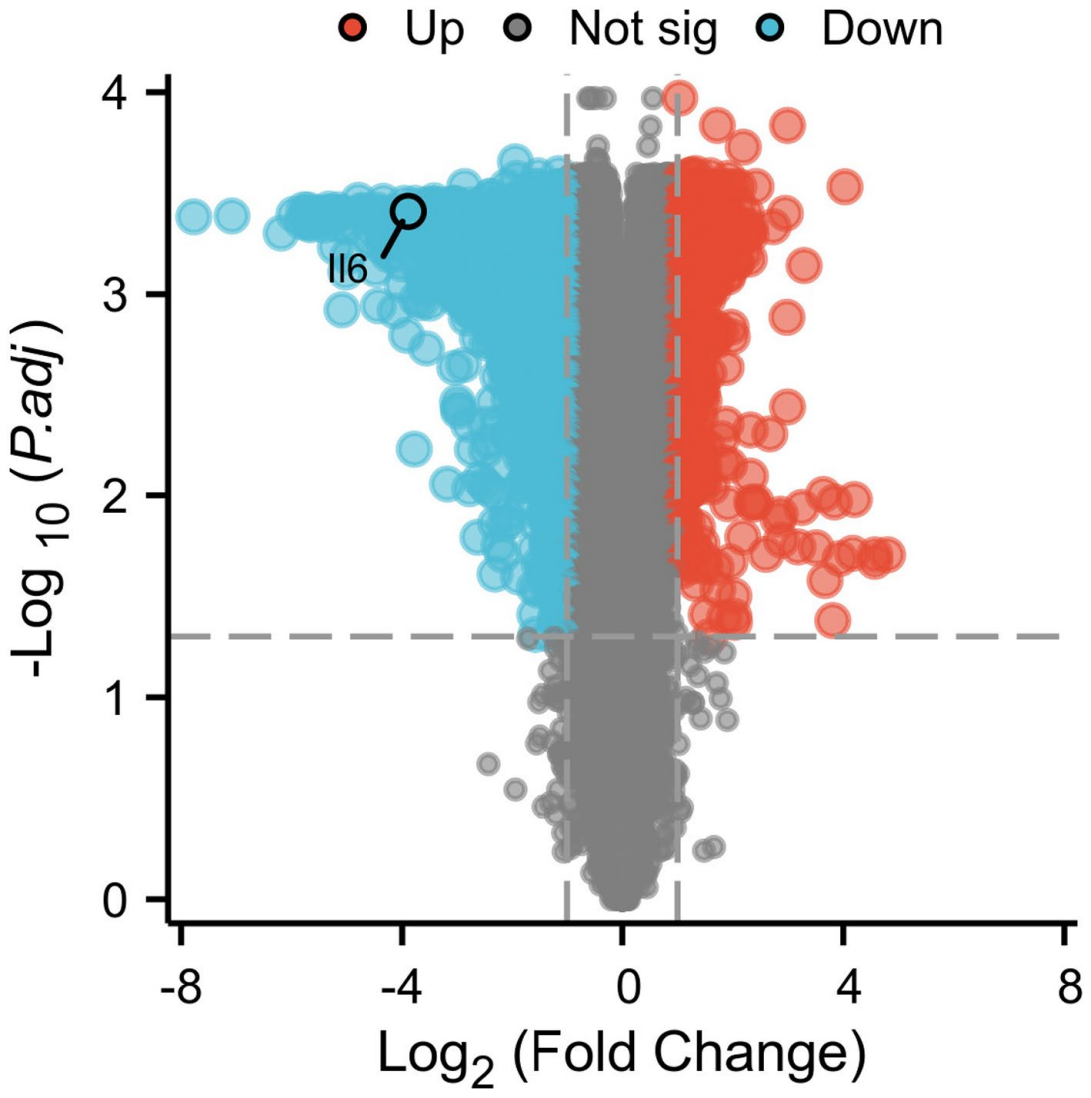
Volcano plot of differentially expressed genes (DEGs).

The genes in the spinal cord tissues of mice from the Control and SCI groups were analyzed with GEO 2R to identify differentially expressed genes (DEGs). Xian Tao Academic was used to create a volcano plot, shown in Figure 7, illustrating 1,229 DEGs: 382 up-regulated and 847 down-regulated.

**Figure 8:**
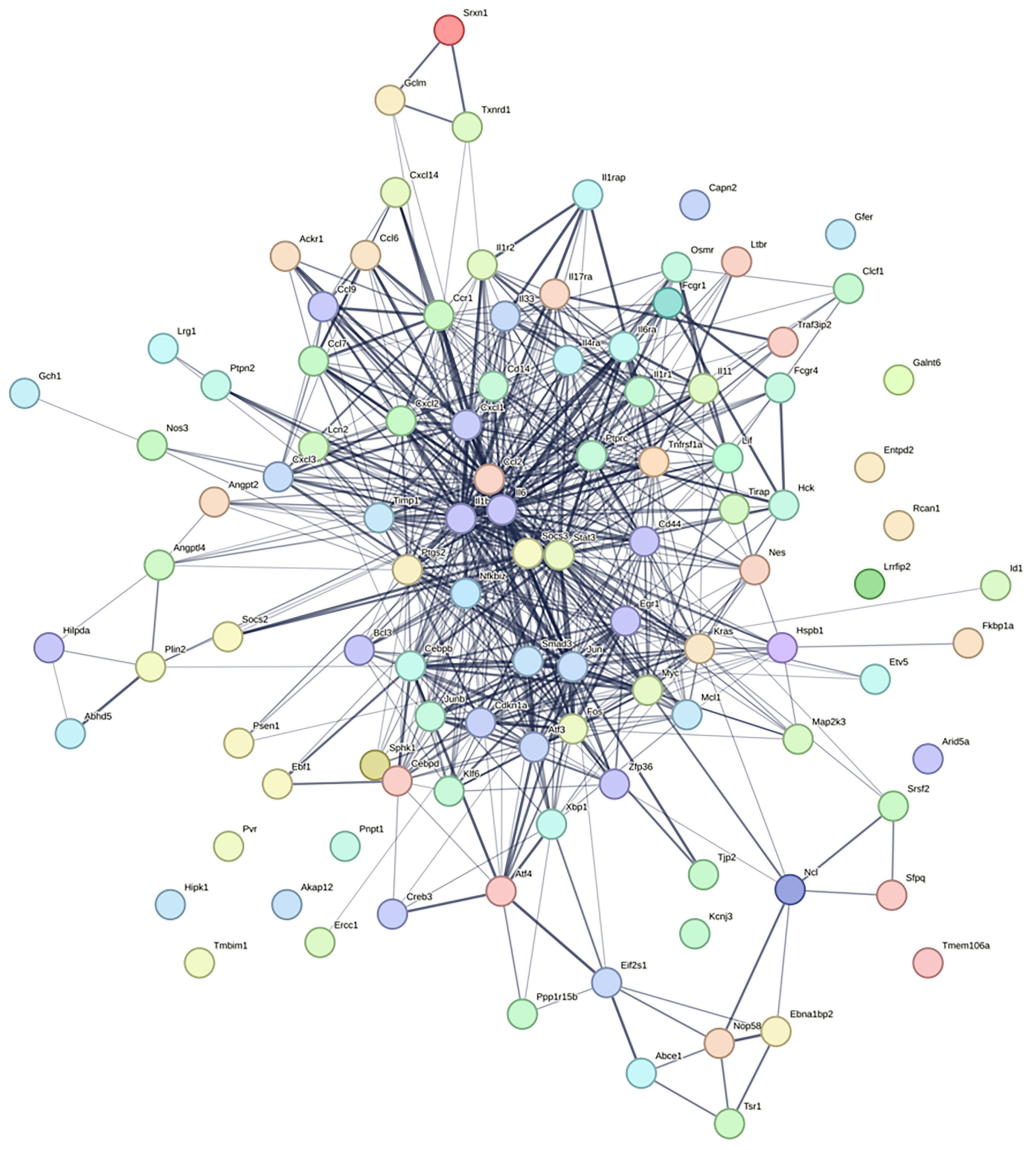
Protein–Protein Interaction (PPI) network analyzed using STRING.

**Figure 9:**
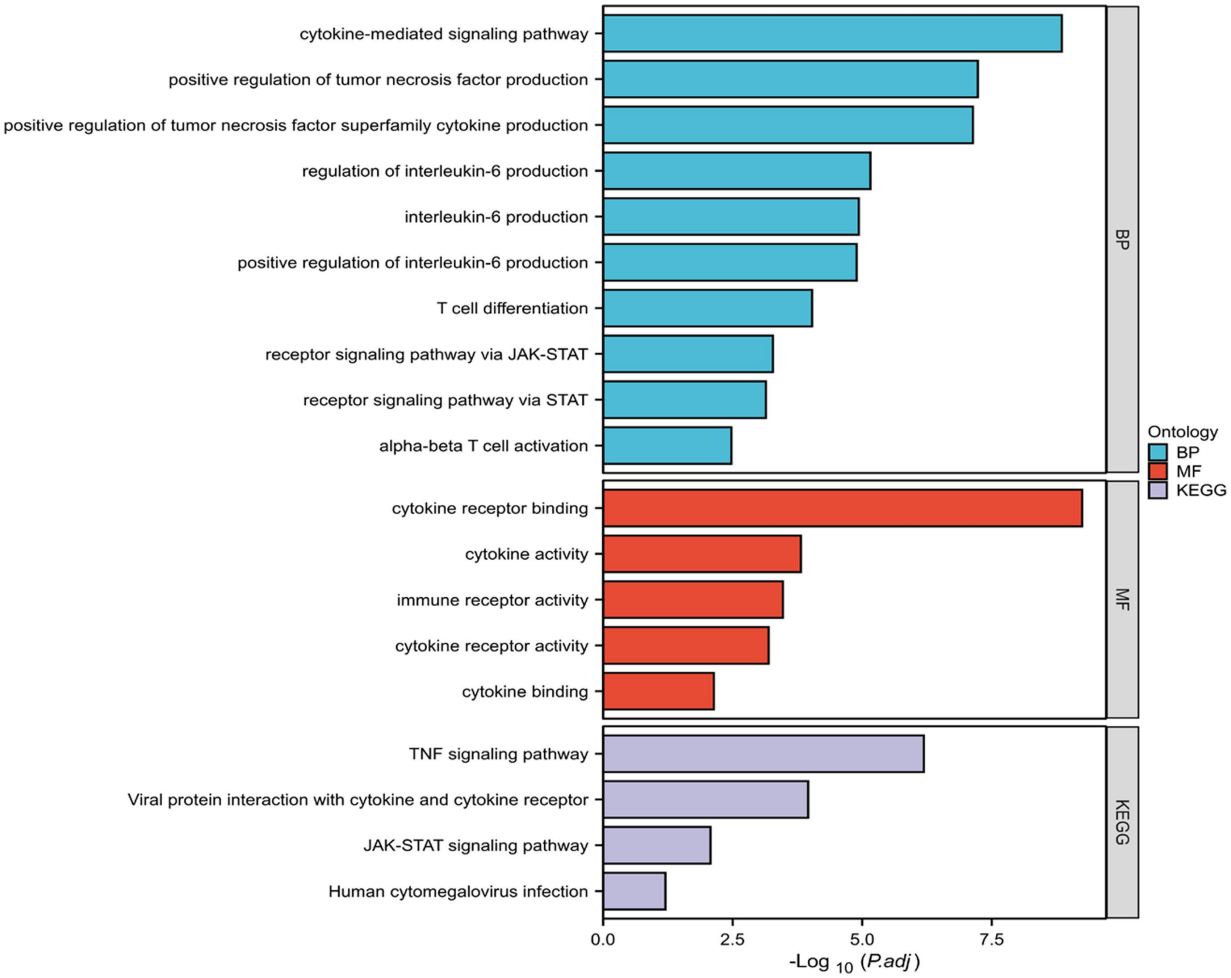
Results of gene ontology (GO) enrichment analysis.

The GO enrichment analysis revealed that the DEGs were primarily involved in biological processes like “cytokine-mediated signaling,” “T-cell differentiation,” and “IL-6 production and regulation” (Figure 8). For molecular functions, they were enriched in “cytokine receptor binding”, “cytokine activity”, and related activities (Figure 8). KEGG pathway analysis indicated that the DEGs were significant in pathways such as “tumor necrosis factor signaling”, “viral protein interactions with cytokines”, “JAK–STAT signaling”, and “human cytomegalovirus infection” (Figure 9).

The Protein–Protein Interaction (PPI) network was analyzed using STRING and visualized with Cytoscape. The CytoHubba plugin identified the top 10 key genes: *Il-6*, *Il1b*, *Jun*, *Ccl2*, *Fos*, *Stat3*, *Ptgs2*, *Kras*, *Myc*, and *Egr1*. Core pro-inflammatory factors like *Myc*, *Ccl2*, *Jun*, *Il1b*, and *Il6* are vital in inflammation. *Myc* and *Jun* regulate inflammation-related genes, *Ccl2* recruits immune cells, and *Il1b* and *Il6* are key pro-inflammatory cytokines that drive the inflammatory response.

**Figure 10:**
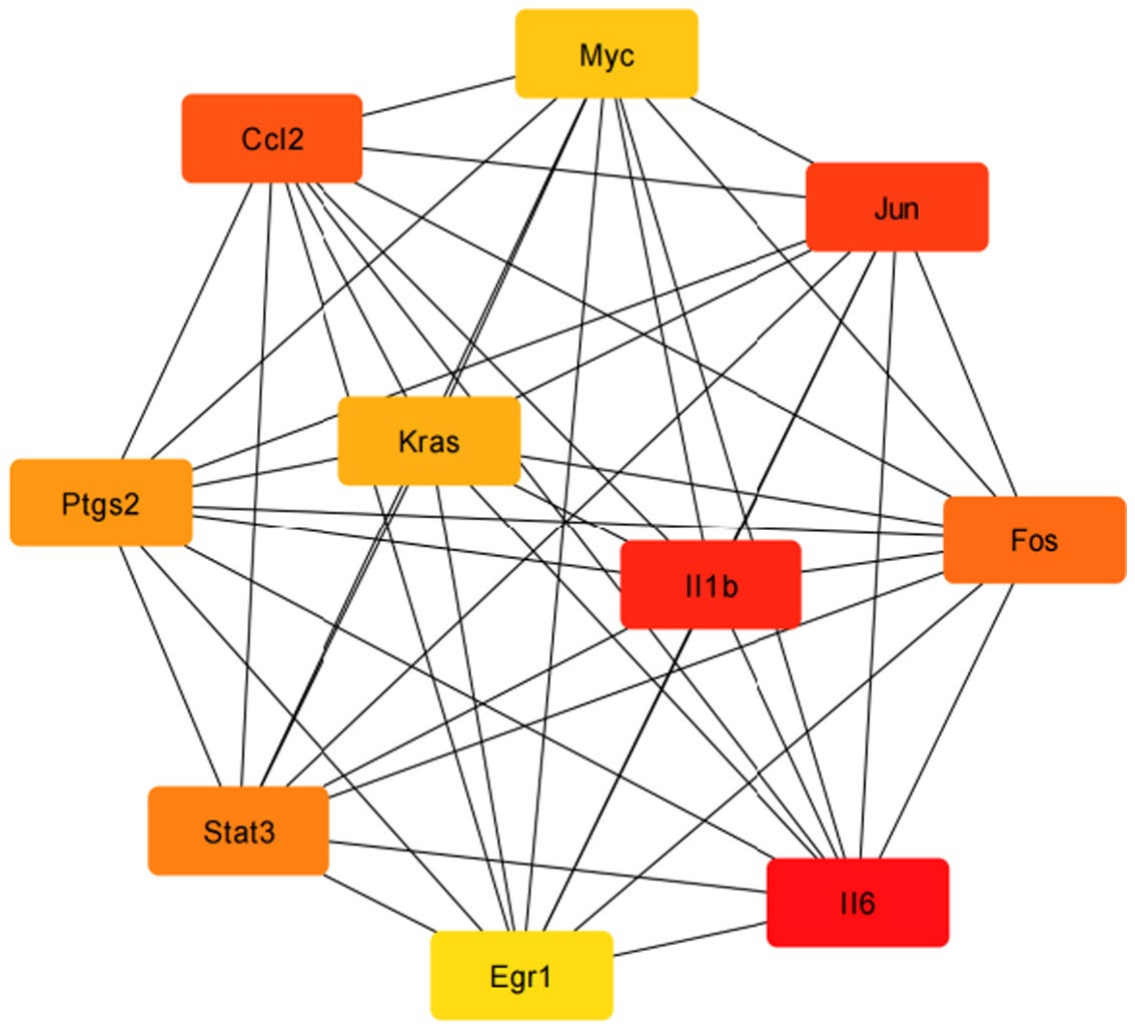
Visual analysis of the PPI network.

Inflammatory regulation network: Proteins form a complex network to regulate inflammation. *Il6* and *Il1b* work together to enhance inflammatory factor production. *Stat3*, involved in the IL-6 pathway, controls inflammation-related gene expression, thus modulating the inflammatory response. See Figures 8, 10 for details.

(1) IL-6: IL-6 is a key player in inflammation, influencing secondary injury and nerve repair after SCI. Its levels spike quickly post-injury, reflecting acute inflammation. Prolonged elevation can worsen neuronal damage and promote glial scar formation. During rehabilitation, IL-6 levels gradually decrease, indicating reduced inflammation and improved nerve function. Electrical stimulation, a vital rehab technique, helps lower IL-6 and TNF-*α*, reducing injury and aiding repair. Tracking IL-6 changes helps evaluate the effectiveness of electrical stimulation in controlling inflammation and enhancing nerve recovery, making IL-6 an important biomarker for inflammation assessment.
(2) Superoxide Dismutase (SOD): After SCI, free radicals increase, harming neuron membranes and accelerating cell death, resulting in neurodegeneration. SOD, an essential free radical scavenger, shields nerve cells from oxidative damage. Following SCI, SOD activity decreases, worsening the injury. Therefore, SOD levels are crucial for assessing free radical clearance and neurological recovery.

### 2.4 Termination criteria

The experiment will end if the participant faces severe complications like pressure ulcers, pulmonary infections, or deep vein thrombosis, or if they leave the hospital for personal reasons or withdraw from the study.

### 2.5 Quality control of evaluation indicators

To reduce evaluator bias, a rehabilitation specialist will administer electrical stimulation therapy. Evaluators will be blinded to group assignments and will consistently assess each participant before and after treatment.

### 2.6 Statistical methods

The sample size was calculated based on the primary outcome of change in SCIM III score at 8 weeks. A minimal clinically important difference of 8 points was selected and the standard deviation was estimated at 15. With a two-sided *α* of 0.05 and statistical power (1 − *β*) of 80%, considering for a 10% dropout rate, the final sample size was 25. The participants were randomly divided into experimental group (*n* = 13) and control group (*n* = 12).

Statistical analysis will use SPSS version 27.0. For normally distributed data, paired and independent *t*-tests will compare within and between groups, respectively, with results as mean ± standard deviation. Non-normally distributed data will use rank-sum tests: Wilcoxon signed-rank for paired data and Wilcoxon–Mann–Whitney for between-group comparisons, shown as median (P25, P75). Categorical variables will be analyzed with chi-square or Fisher’s exact tests, and repeated measures with ANOVA. A *p*-value of <0.05 will denote statistical significance.

## 3 Results

### 3.1 Comparison of general demographic data of study participants

**Figure 11:**
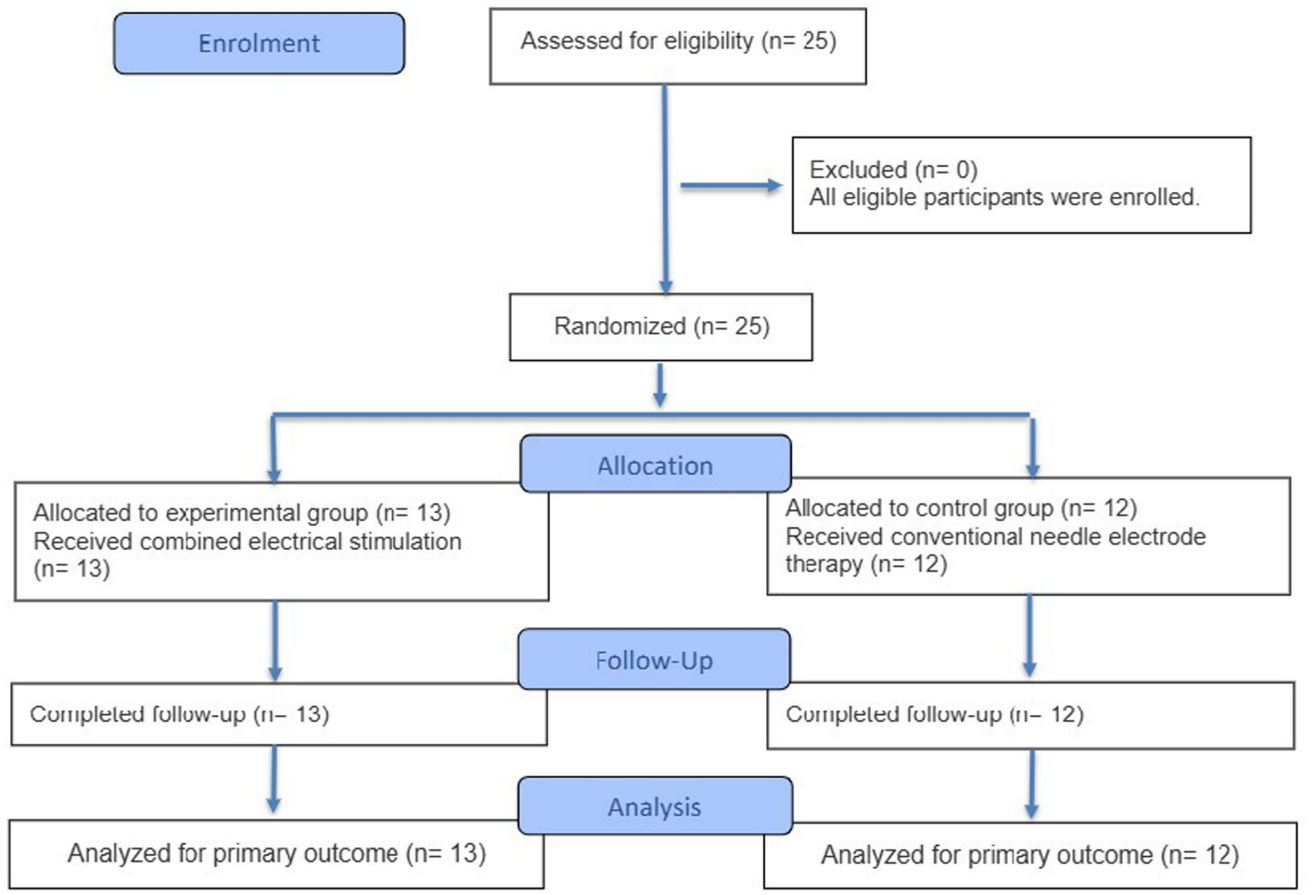
CONSORT flow diagram. Flow diagram of the progress through the phases of a randomised trial of two groups (that is, enrolment, intervention allocation, follow-up, and data analysis).

Participants were randomly divided into an experimental group (*n* = 13) and a control group (*n* = 12). The experimental group had an average age of 47.85 years and an injury duration of 41 days, while the control group averaged 52.25 years and 36 days of injury duration. Detailed demographics are in Table 2. The research was conducted and reported in strict accordance with the Standards of Reporting Trials (CONSORT) statement. The full process of participant recruitment, eligibility screening, random allocation, follow-up, and final data analysis is presented in Figure 11.

**Table 2:** Comparison of general demographic data between the two groups (x̄ ± s).

Group	Experimental group (*n* = 13)	Control group (*n* = 12)	*p* Value
Age (years)	47.85 ± 10.98	52.25 ± 6.52	0.24
Weight (kg)	64.92 ± 5.95	66.33 ± 6.69	0.624
Height (cm)	171.38 ± 6.65	172.75 ± 7.10	0.582
Duration of injury (days)	41.00 (34.16, 69.99)	36.00 (31.80, 64.70)	0.735

Table shows no significant differences in age, height, weight, or injury duration between the experimental and control groups (*p* > 0.05), indicating comparable baseline characteristics for valid comparisons.

### 3.2 Comparison of pre- and post-treatment FIM scores between groups

**Figure 12:**
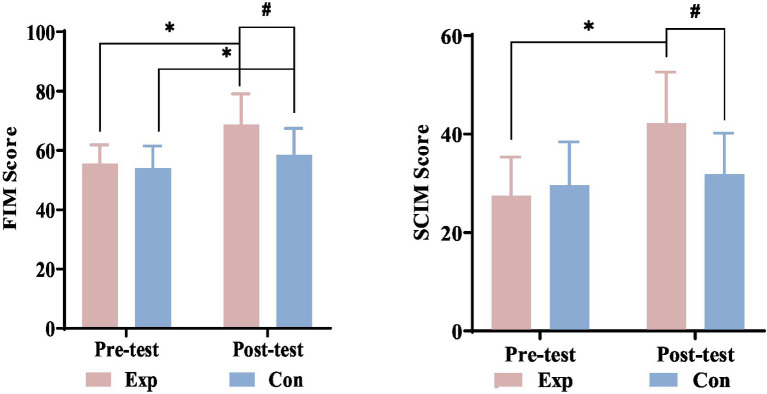
Comparison of FIM and SCIM-III scores before and after treatment between the experimental and control groups. ^*^Denotes significant within-group difference, *p* < 0.05; ^#^denotes significant between-group difference, *p* < 0.05. Exp, experimental group; Con, control group.

After 8 weeks, both groups had significantly better FIM scores, but only the experimental group showed significant SCIM-III score improvements. Initially, there were no significant differences between the groups’ FIM and SCIM-III scores. Post-treatment, the experimental group had significantly greater functional independence improvements than the control group. See Figure 12 for detailed statistics.

### 3.3 Comparison of pre- and post-treatment sensory scores between groups

After 8 weeks of electrical stimulation, both the experimental and control groups showed improved sensory function scores. A repeated measures ANOVA assessed the interventions’ impact over time. Due to a significant Mauchly’s sphericity test (*p* < 0.05), indicating a sphericity violation, a corrected variance analysis was conducted. The findings showed a significant time effect (*P*_time_ < 0.05), with ASIA sensory scores for both groups increasing notably by weeks 2, 4, 6, and 8 of treatment. The time × group interaction was also significant (*P*_time × group_ <0.001), indicating the scores differed significantly between the groups over time.

**Figure 13:**
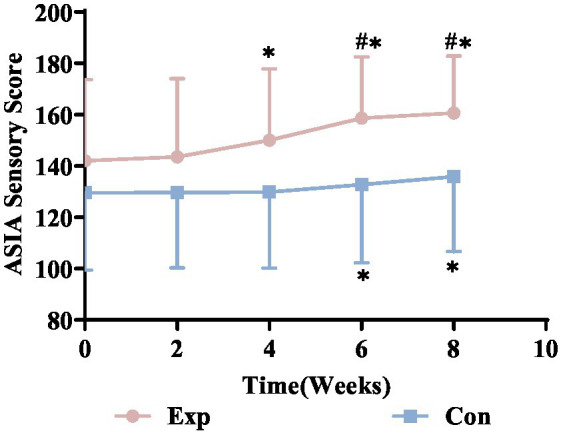
ASIA sensory scores trends for experimental vs. control groups. ^*^Denotes significant within-group difference, *p* < 0.05; ^#^denotes significant between-group difference, *p* < 0.05. Exp, experimental group; Con, control group.

In the experimental group, significant changes started at week 4, while in the control group, they began at week 6. Initially, there were no significant differences between the groups (*p* > 0.05), but by week 6, significant differences appeared (*p* < 0.05), showing that the experimental group recovered sensory function faster and more effectively. Detailed statistics are in Figure 13.

### 3.4 Motor function scores comparison pre- and post-treatment in both groups

#### 3.4.1 ASIA motor function score

After 8 weeks of intervention, both the experimental and control groups significantly improved their ASIA motor function scores. Repeated measures ANOVA assessed the interventions’ effects over time. Due to a *p* value <0.05 from Mauchly’s test, indicating a violation of sphericity, a corrected variance analysis was conducted. The results showed a significant time effect (*P*_time_ < 0.05), with ASIA motor scores for both groups increasing notably at weeks 2, 4, 6, and 8 post-treatment. Additionally, a significant time × group interaction (*P*_time × group_ <0.05) indicated that the scores between the groups diverged significantly over time.

**Figure 14:**
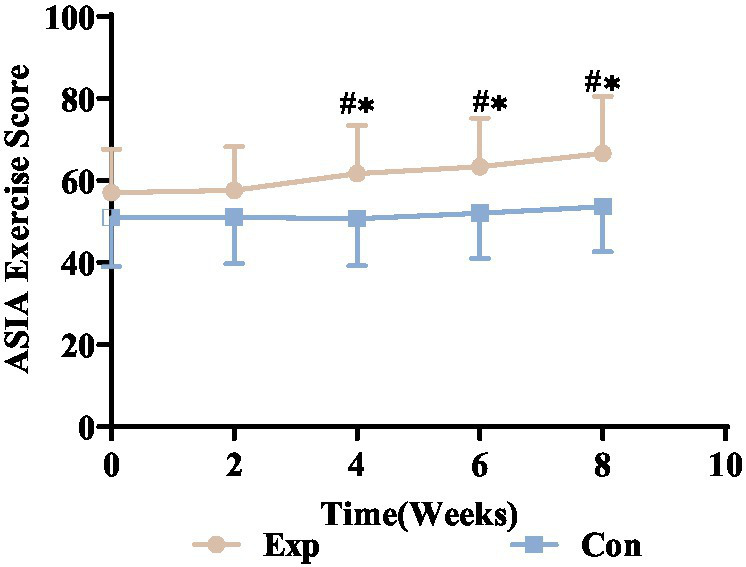
ASIA motor scores trends for experimental and control groups. ^*^Denotes significant within-group difference, *p* < 0.05; ^#^denotes significant between-group difference, *p* < 0.05. Exp, experimental group; Con, control group.

From week 4, the experimental group demonstrated significant motor function improvement, unlike the control group, which showed no notable changes at any point. Initially, both groups had similar ASIA motor scores, but the experimental group improved significantly faster and more prominently, as shown in Figure 14.

#### 3.4.2 Lower limb surface EMG RMS

After 8 weeks of treatment, statistical analyses showed no significant differences in muscle tone during passive movements between the experimental and control groups, indicating no increased spasticity (*p* > 0.05). However, during active movements, the experimental group showed a significant increase in RMS values for the vastus lateralis, rectus femoris, and vastus medialis muscles compared to pre-treatment (*p* < 0.05). In contrast, while the tibialis anterior and gastrocnemius muscles also showed increased RMS values during active movements, these changes were minimal and not statistically significant (*p* > 0.05).

**Figure 15:**
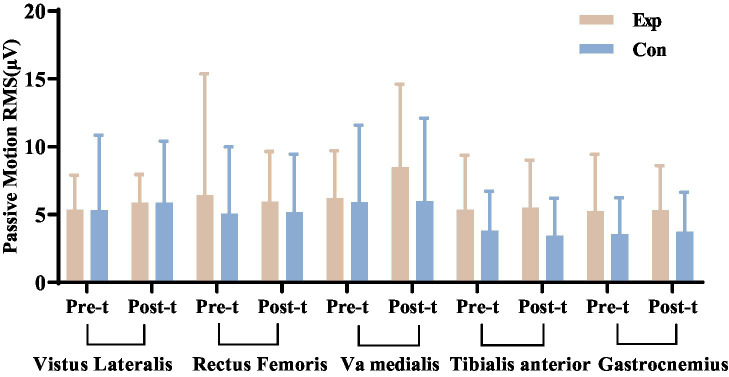
Comparison of the RMS values of passive movement for key lower limb muscles, assessed before and after treatment in both the experimental and control groups. ^*^Denotes significant within-group difference, *p* < 0.05; ^#^denotes significant between-group difference, *p* < 0.05. Exp: experimental group; Con: control group.

**Figure 16:**
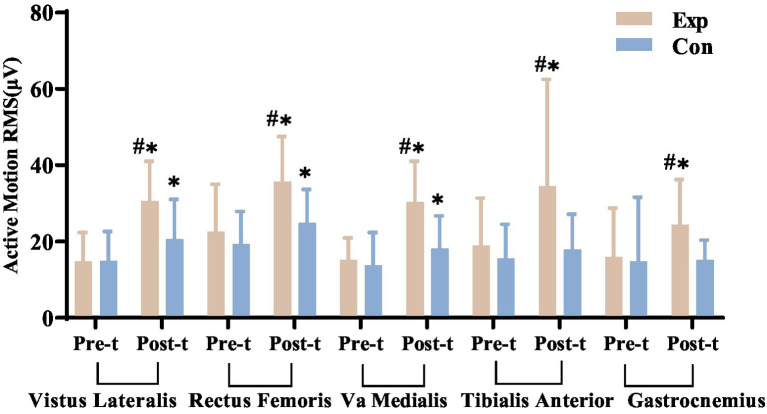
Comparison of pre- and post-treatment RMS values for key lower limb muscles in experimental and control groups. ^*^Denotes significant within-group difference, *p* < 0.05; ^#^denotes significant between-group difference, *p* < 0.05.

Before treatment, no significant differences were found in passive and active movements between the experimental and control groups. After treatment, passive movement differences remained insignificant, but active movement showed significant differences in RMS values for key lower limb muscles, with the experimental group showing greater improvements. This suggests enhanced muscle strength in the experimental group post-treatment. Detailed results are in Figures 15, 16.

#### 3.4.3 Lower limb muscle surface iEMG

After 8 weeks of treatment, both the experimental and control groups showed significant increases in iEMG values for the vastus lateralis, rectus femoris, vastus medialis, and tibialis anterior muscles during movement (*p* < 0.05). However, the gastrocnemius muscle in the control group showed no significant change from baseline.

**Figure 17:**
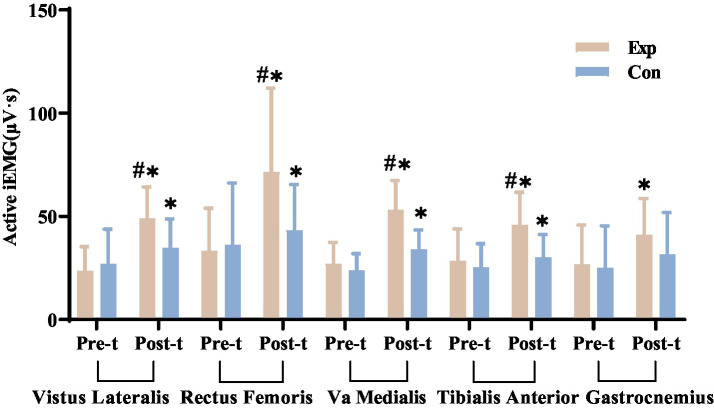
Comparison of pre- and post-treatment iEMG values of key lower limb muscles in experimental and control groups. ^*^Denotes significant within-group difference, *p* < 0.05; ^#^denotes significant between-group difference, *p* < 0.05. Exp, experimental group; Con, control group.

Initial comparisons showed no significant differences in passive and active movements between the groups (*p* > 0.05). After treatment, passive movements remained similar (*p* > 0.05), but the experimental group had significantly different iEMG values for several muscles during active movement compared to the control group (*p* < 0.05). This suggests that after 8 weeks, the experimental group had improved neuromuscular function, with increased motor unit recruitment. Detailed results are in Figure 17.

### 3.5 Comparison of blood biochemical indicators pre- and post-treatment in both groups

After 8 weeks of treatment, both the experimental and control groups showed significant increases in SOD levels and decreases in IL-6 levels compared to their baseline measurements (*p* < 0.05).

**Figure 18:**
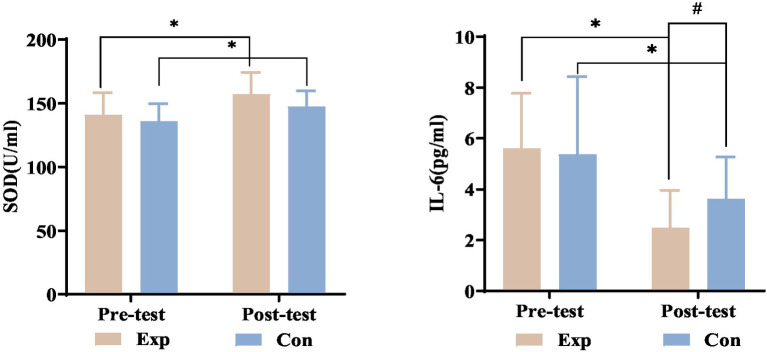
SOD and IL-6 levels before and after treatment in both experimental and control groups. ^*^Denotes significant within-group difference, *p* < 0.05; ^#^denotes significant between-group difference, *p* < 0.05. Exp, experimental group; Con, control group.

Before treatment, there were no significant differences in SOD and IL-6 levels between the experimental and control groups (*p* > 0.05). After treatment, IL-6 levels significantly increased in the experimental group compared to the control group (*p* < 0.05), indicating a greater reduction in IL-6. SOD levels remained similar between the groups post-treatment (*p* > 0.05). See Figure 18 for detailed statistics.

## 4 Discussion

Spinal cord injuries (SCI) lead to functional impairments below the injury site, often caused by spinal diseases or trauma. Without timely treatment, they can cause death from respiratory failure or infections. SCI disrupts neural pathways, resulting in permanent disability, psychological distress, and economic strain. While the central nervous system (CNS) has some ability to regenerate, this capacity is limited compared to the peripheral nervous system and decreases with age (25). Moreover, SCI initiates a cascade of intricate pathophysiological alterations that result in a “microenvironmental imbalance.” This imbalance exacerbates and accelerates the progression of SCI, thereby impeding neural regeneration and functional recovery (26). SCI presents significant treatment challenges and is a major obstacle in neurological disorders. Incomplete SCI is more common than complete SCI, and patients with incomplete injuries have better recovery prospects due to some remaining neural connections. Thus, this study focused on patients with incomplete SCI. Early rehabilitation is crucial for SCI patients, so the study included those 1–6 months post-injury to avoid the spinal shock phase, which lasts 1–4 weeks and causes total motor and reflex loss, increasing complication risks. This timing aimed to preserve neural tissue and improve recovery without spinal shock affecting evaluations (27).

### 4.1 Benefits of integrated electrical stimulation therapy

A review of the literature reveals that current electrical stimulation therapies for SCI primarily target the head (28), spinal cord (29), or peripheral nerves. Nevertheless, the comprehensive therapeutic outcomes for SCI patients remain constrained, necessitating advancements in functional recovery. It is evident that there is a critical need to investigate intervention strategies that can more effectively leverage residual neural connections to augment functional recovery and optimize the quality of life for individuals with SCI. Recent studies have demonstrated that the integration of electrical stimulation at multiple sites holds promise, yielding significant therapeutic benefits in the treatment of SCI. For instance, Pan Ran (30) and colleagues applied a combination of Du Mai and back Shu acupoints with peripheral electrical stimulation. These acupoints are located near spinal nerves and their branches. Electrical stimulation here can activate spinal nerve roots and the spinal dura mater, promoting nerve regeneration and improving the environment around injured nerves. Combining peripheral electrical stimulation sends nerve impulses to the brain, which then activates muscles through descending pathways, significantly improving sensory function and daily activities in SCI patients. Research shows that multi-site electrical stimulation is beneficial, aligning with traditional Chinese medicine’s holistic approach. Treatments should consider a comprehensive strategy beyond just the spinal cord for better functional recovery. Few studies have examined combined electrical stimulation of the head, spinal cord, and peripheral nerves. This study implemented an 8-week regimen targeting these areas for patients with incomplete SCI, suggesting that head stimulation activates descending pathways and peripheral stimulation.

### 4.2 Data mining to determine the electrical stimulation sites

In this study, we used data mining to analyze literature on acupuncture for SCI, identifying the Jiaji acupoints as the best sites for electrical stimulation. These acupoints, located near the spine, are closely linked to the spinal cord and nerves. Stimulating them can modulate neural activity, promote neuron regeneration, enhance neural plasticity, and improve signal transmission between the spinal cord and brain, offering significant clinical benefits.

The data mining-based acupoint selection method enhances the precision of electrical stimulation for incomplete SCI and advances the modernization of acupuncture. By merging traditional Chinese medicine with modern technology, it fosters innovation in SCI rehabilitation.

### 4.3 Bioinformatics analysis to identify key genes in SCI

This study used bioinformatics to identify key genes involved in inflammation after SCI from gene expression databases. The crucial genes identified were *Il6*, *Il1b*, *Jun*, *Ccl2*, *Fos*, *Stat3*, *Ptgs2*, *Kras*, *Myc*, and *Egr1*, which regulate inflammation-related gene expression, recruit immune cells, and stimulate inflammation. Tracking these gene expressions helps us understand the impact of combined electrical stimulation on SCI-related inflammation. *Il6* and *Il1b* increase inflammatory factors, while *Stat3*, part of the IL-6 pathway, regulates inflammation-related genes. This insight aids in developing anti-inflammatory treatments and improving electrical stimulation protocols for SCI.

### 4.4 Advancements in electrical stimulation techniques

Currently, electrical stimulation therapy abroad can be categorized based on the depth of electrode placement into Transcutaneous Electrical Nerve Stimulation (TENS) (31), Percutaneous Electrical Nerve Stimulation (PENS) (32), and implantable neuromodulation techniques (15). TENS is non-invasive, with electrodes placed on the skin, but it struggles to stimulate deep muscles and nerves effectively due to skin conductivity issues. Conversely, PENS places electrodes under the skin, enabling deeper stimulation. Epidural Spinal Cord Stimulation (eSCS) is a prevalent treatment for SCI, involving surgically implanted electrodes in the epidural space to directly stimulate the spinal cord. Additionally, low-frequency TENS post-SCI can enhance postural control and reactivate spinal cord networks (31).

Implantable electrodes provide superior accuracy and therapeutic benefits compared to TENS but are limited by surgical risks, higher costs, and infection risks. PENS delivers deeper stimulation without surgical implantation, minimizing risks and costs. In China, Electro Acupuncture (EA) is similar to PENS and is well-researched. EA uses acupuncture needles and electrical currents for therapeutic effects, but acupuncture on meridian points carries a 1.3% risk of nerve injury from improper needle placement (33, 34). Ultrasound, a safe and non-invasive imaging method, is extensively used to assess skeletal muscles and nerves in the limbs and trunk, providing clear visualization of needle insertion points and surrounding tissues (26), This study enhances electrical stimulation by using acupuncture needles for precise, real-time needle placement, minimizing nerve and structural damage. This method avoids the risks of implantable electrodes while maintaining effective stimulation strength and targeting. We used portable ultrasound to measure the skin-to-nerve distance for precise needle insertion during epidural and sciatic nerve procedures, ensuring accurate stimulation and preventing nerve damage. This study aimed to examine the effects of combined electrical stimulation on sensory, motor function, and activity in patients with incomplete SCI, and to explore its underlying mechanisms.

### 4.5 Impact of electrical stimulation on Independence in incomplete SCI patients

Patients with SCI often experience dysfunction in their bodies and limbs, requiring long-term rehabilitation. The FIM measures functional disability and independence in six areas, with higher scores indicating better function and quality of life. The SCIM-III focuses on self-care, respiratory and sphincter control, and mobility for SCI patients, providing a more detailed assessment than the FIM. After 8 weeks of intervention, both groups showed significant improvement in FIM scores. The experimental group’s SCIM-III score increased significantly, while the control group’s did not. Pre-treatment scores were similar, but post-treatment scores differed significantly, showing that combined electrical stimulation significantly improved functional activities and life skills in patients with incomplete SCI compared to the control group. These findings are consistent with previous research (35), we propose that improved functional activity is connected to restored sensory and motor functions, likely due to enhanced neural pathway re-establishment and axonal regeneration, leading to greater functional independence and self-care.

### 4.6 Effects of electrical stimulation on sensory function in incomplete SCI patients

The spinal cord’s dorsal horn houses sensory axons that relay signals to the brain via complex pathways. These axons are prone to damage from external trauma, with spinal cord injuries often affecting the sensory axons connecting the dorsal root ganglia to the cerebral cortex (36). When sensory conduction pathways are disrupted, the brain cannot receive signals from sensory receptors, leading to deficits like numbness and tingling, which greatly affect the patient’s quality of life. Assessing these pathways is crucial for evaluating SCI severity and recovery. This study used the ASIA sensory score to evaluate sensory function at 28 key body points, with higher scores indicating better function (21). Results indicated significant improvements in ASIA sensory scores for both groups post-treatment. The experimental group showed improvement from week 4, while the control group improved from week 6, suggesting that combined electrical stimulation enhances sensory function faster than needle electrode therapy. Before treatment, sensory scores were similar between groups (*p* > 0.05). However, from week 6, the experimental group showed significantly better results (*p* < 0.05). This indicates that combined electrical stimulation enhances sensory function and speeds up recovery more effectively than needle electrode therapy.

### 4.7 Effects of electrical stimulation on motor function in incomplete SCI patients

#### 4.7.1 Impact of electrical stimulation on motor scores in incomplete SCI patients

After SCI, disrupted neural pathways hinder signal transmission from the brain to upper motor neurons, causing motor dysfunction. The integrity of nerve fibers is vital for neuromuscular function. Research indicates that SCI increases the expression of Id2, which negatively affects myelin basic protein, leading to ongoing demyelination (37). In cases of incomplete SCI, demyelination impairs motor axon signal conduction, worsening motor dysfunction and daily life. Thus, restoring motor function is essential. This study used the ASIA motor score to evaluate motor function, showing a 16% improvement in the experimental group from pre-treatment to week 8. Significant improvements were observed from week 4 in the experimental group, while the control group showed minimal changes. Initially, motor scores were similar between groups, but by week 8, the experimental group had superior improvement. This indicates that combined electrical stimulation is more effective and faster than needle electrode therapy for improving motor function in incomplete SCI patients.

#### 4.7.2 Effects of combined electrical stimulation on lower limb muscle EMG in incomplete SCI patients

Although several studies have confirmed the reliability and validity of the ASIA scoring system (38, 39), The ASIA motor score relies on therapists’ manual muscle strength assessments, leading to subjective bias. In contrast, sEMG uses surface electrodes to non-invasively and quantitatively measure bioelectrical activity from spinal motor neurons, indicating muscle activity and function. This method is commonly used in neuromuscular testing and rehabilitation (40). We used sEMG to assess muscle strength changes in SCI patients, focusing on key lower limb muscles affected by tetraplegia and paraplegia. We measured RMS and iEMG during passive and active movements, finding both correlated with muscle strength and function. Post-treatment, the experimental group showed significant increases in RMS and iEMG for all targeted muscles during active movements (*p* < 0.05), while the control group showed increases in some muscles (*p* < 0.05). Initially, there were no significant differences between groups (*p* > 0.05), but after 8 weeks, the experimental group had significantly greater improvements (*p* < 0.05).

### 4.8 Effects of electrical stimulation on blood markers in incomplete SCI patients

SCI can cause an initial injury and subsequent secondary damage, which involves oxidative stress, inflammation, additional cell death, demyelination, and axonal degeneration (41). Secondary injury can be more harmful than the primary injury, significantly contributing to neuronal death and neurological deficits. Researchers have long considered targeting secondary injury as a promising therapeutic strategy for functional recovery. Preventing secondary injury and offering neuroprotection can help preserve tissue function, and even minimal neuroprotective effects can greatly enhance recovery after SCI (42). We aimed to investigate physiological mechanisms by examining changes in blood biochemical markers in SCI patients before and after treatment.

#### 4.8.1 Impact of combined electrical stimulation on SOD in incomplete SCI patients

Following SCI, mitochondrial dysfunction produces excessive reactive oxygen species (ROS) and free radicals, disrupting antioxidant balance and causing lipid peroxidation. These ROS interact with polyunsaturated fatty acids in cell membranes, altering permeability and resulting in necrosis and spinal cord damage. Intracellular antioxidants such as SOD and glutathione peroxidase (GSH-Px) play a protective role (43). SOD is vital for reducing ROS and protecting cells from oxidative damage. Our study showed that after 8 weeks, SOD levels rose significantly in both experimental and control groups compared to baseline (*p* < 0.05), with significant differences between the groups after treatment (*p* < 0.05). This suggests that both combined electrical stimulation and needle electrode therapy can increase SOD levels, with electrical stimulation being more effective. We conclude that electrical stimulation may reduce oxidative stress-related damage after SCI by increasing SOD levels, protecting neural pathways, and supporting neurological recovery. It may also regulate sympathetic nerves, reducing vertebral artery ischemia and ROS production, thus maintaining SOD levels.

#### 4.8.2 Impact of combined electrical stimulation on IL-6 in incomplete SCI patients

Inflammation complicates recovery in both acute and chronic stages of SCI by worsening initial damage during the secondary injury phase. Autonomic nervous system dysregulation and secondary complications like infections can prolong chronic inflammation, exacerbating SCI (44). Managing chronic inflammation is crucial for improving the health and quality of life of SCI patients, as it can lead to complications, pain, and depression, affecting recovery and survival. The study focused on IL-6, a pro-inflammatory cytokine associated with neurological trauma. Results showed a significant decrease in IL-6 levels after treatment in both experimental and control groups (*p* < 0.05), with no significant difference between them (*p* > 0.05). Reduced level of IL-6 may means that inflammation is suppressed, neurotoxic effects are reduced, and secondary pathological processes after SCI are alleviated, which has significant clinical significance for judging the prognosis of patients with incomplete SCI. This suggests that both electrical stimulation and needle electrode therapy effectively reduce inflammation in patients with incomplete SCI, likely influenced by the micturition reflex centers in the sacral spinal cord. SCI frequently interrupts this connection, resulting in irregular micturition reflexes and neurogenic bladder. Most study participants needed indwelling catheters for urination assistance. Prolonged catheter use can lead to urethral injuries and urinary tract infections, with incidence rates in SCI patients between 7.41 and 62% (45). No severe urinary tract infections were reported, but potential infection risks may have influenced inflammation and study results. Pandemic-related recruitment issues and participant drop-outs resulted in a small sample size, possibly affecting the findings.

The study demonstrates that combined electrical stimulation and needle electrode therapy improve sensory and motor functions, functional independence, and biochemical markers in patients with incomplete SCI, with electrical stimulation being more effective. It enhances quality of life and may increase antioxidant capacity, aiding recovery by protecting neural pathways and muscles. No adverse events were reported, indicating its safety and effectiveness, supporting its broader use in SCI rehabilitation.

### 4.9 Limitation and advice

We find that the two therapies synergize to enhance overall treatment efficacy. But our research still has several limitations. This study was a short-term intervention of 8-weeks, and there was no long-term follow-up of patients. It is suggested that a multicenter randomized clinical trial can be conducted in follow-up studies. Given to the modest sample size, and patients had different SCI segments and courses, the findings warranting validation in larger populations. This suggests that subsequent studies can expand the sample size, recruit more patients with incomplete SCI for the study, and differentiate the injury level of patients to refine the grouping, and further observe the efficacy of combined electrical stimulation in the treatment of incomplete SCI.

## 5 Conclusion

An 8-week regimen of combined electrical stimulation and needle electrode therapy can enhance sensory and motor functions, boost functional independence, and aid recovery in patients with incomplete SCI by increasing antioxidants and reducing chronic inflammation.

Combined electrical stimulation is more effective than needle electrode therapy and has no adverse events, making it a safe and effective rehabilitation method for patients with incomplete SCI.

## Data Availability

The datasets presented in this study can be found in online repositories. The names of the repository/repositories and accession number(s) can be found in the article/supplementary material.
